# Supplementary material for: The application of deep learning in early enamel demineralization detection
Source: PeerJ. 2025 Jan 2;13:e18593. doi: 10.7717/peerj.18593 (PMC11700490; doi:10.7717/peerj.18593)
Supplement: Supplemental Information 1 [file peerj-13-18593-s001.docx]

**Recognition of each tooth position by the test set**

| tooth position | | TP | FP | TN | FN | NPV | PPV | SEN | SPEC | IoU | F1-score |
| --- | --- | --- | --- | --- | --- | --- | --- | --- | --- | --- | --- |
| 11,12,21,22 | junior1 | 23 | 7 | 49 | 1 | 0.980 | 0.767 | 0.958 | 0.875 | 0.5518 | 0.852 |
|  | junior2 | 23 | 7 | 49 | 1 | 0.980 | 0.767 | 0.958 | 0.875 | 0.6026 | 0.852 |
|  | DL-assisted junior1 | 24 | 4 | 52 | 0 | 1.000 | 0.857 | 1.000 | 0.929 | 0.7536 | 0.923 |
|  | DL-assisted junior2 | 24 | 3 | 53 | 0 | 1.000 | 0.889 | 1.000 | 0.946 | 0.6406 | 0.941 |
|  | AI | 24 | 7 | 49 | 0 | 1.000 | 0.774 | 1.000 | 0.875 | 0.6418 | 0.873 |
| 13,23 | junior1 | 9 | 4 | 27 | 0 | 1.000 | 0.692 | 1.000 | 0.871 | 0.5925 | 0.818 |
|  | junior2 | 9 | 5 | 26 | 0 | 1.000 | 0.643 | 1.000 | 0.839 | 0.4993 | 0.783 |
|  | DL-assisted junior1 | 9 | 1 | 30 | 0 | 1.000 | 0.900 | 1.000 | 0.968 | 0.7286 | 0.947 |
|  | DL-assisted junior2 | 9 | 1 | 30 | 0 | 1.000 | 0.900 | 1.000 | 0.968 | 0.7881 | 0.947 |
|  | AI | 8 | 0 | 31 | 1 | 0.969 | 1.000 | 0.889 | 1.000 | 0.5338 | 0.941 |
| 24,25,34,35 | junior1 | 12 | 9 | 58 | 1 | 0.983 | 0.571 | 0.923 | 0.866 | 0.6186 | 0.706 |
|  | junior2 | 13 | 7 | 60 | 0 | 1.000 | 0.650 | 1.000 | 0.896 | 0.6188 | 0.788 |
|  | DL-assisted junior1 | 13 | 2 | 65 | 0 | 1.000 | 0.867 | 1.000 | 0.970 | 0.7514 | 0.929 |
|  | DL-assisted junior2 | 13 | 1 | 66 | 0 | 1.000 | 0.929 | 1.000 | 0.985 | 0.7067 | 0.963 |
|  | AI | 13 | 0 | 60 | 7 | 0.896 | 1.000 | 0.650 | 1.000 | 0.6872 | 0.788 |
| 16,26 | junior1 | 6 | 9 | 25 | 0 | 1.000 | 0.400 | 1.000 | 0.735 | 0.6241 | 0.571 |
|  | junior2 | 6 | 16 | 18 | 0 | 1.000 | 0.273 | 1.000 | 0.529 | 0.2155 | 0.429 |
|  | DL-assisted junior1 | 6 | 1 | 33 | 0 | 1.000 | 0.857 | 1.000 | 0.971 | 0.6895 | 0.923 |
|  | DL-assisted junior2 | 6 | 0 | 34 | 0 | 1.000 | 1.000 | 1.000 | 1.000 | 0.7924 | 1.000 |
|  | AI | 5 | 1 | 33 | 1 | 0.971 | 0.833 | 0.833 | 0.971 | 0.6804 | 0.833 |
| 31,32,41,42 | junior1 | 0 | 5 | 75 | 0 | 1.000 | 0.000 | / | 0.938 | / | / |
|  | junior2 | 0 | 4 | 76 | 0 | 1.000 | 0.000 | / | 0.950 | / | / |
|  | DL-assisted junior1 | 0 | 3 | 77 | 0 | 1.000 | 0.000 | / | 0.963 | / | / |
|  | DL-assisted junior2 | 0 | 1 | 79 | 0 | 1.000 | 0.000 | / | 0.988 | / | / |
|  | AI | 0 | 6 | 74 | 0 | 1.000 | 0.000 | / | 0.925 | / | / |
| 33,43 | junior1 | 3 | 1 | 36 | 0 | 1.000 | 0.750 | 1.000 | 0.973 | 0.4642 | 0.857 |
|  | junior2 | 3 | 1 | 36 | 0 | 1.000 | 0.750 | 1.000 | 0.973 | 0.5992 | 0.857 |
|  | DL-assisted junior1 | 3 | 2 | 35 | 0 | 1.000 | 0.600 | 1.000 | 0.946 | 0.7030 | 0.750 |
|  | DL-assisted junior2 | 3 | 1 | 36 | 0 | 1.000 | 0.750 | 1.000 | 0.973 | 0.6773 | 0.857 |
|  | AI | 3 | 2 | 35 | 0 | 1.000 | 0.600 | 1.000 | 0.946 | 0.7730 | 0.750 |
| 34,35,44,45 | junior1 | 3 | 4 | 72 | 1 | 0.986 | 0.429 | 0.750 | 0.947 | 0.6300 | 0.545 |
|  | junior2 | 3 | 5 | 71 | 1 | 0.986 | 0.375 | 0.750 | 0.934 | 0.8220 | 0.500 |
|  | DL-assisted junior1 | 4 | 1 | 75 | 0 | 1.000 | 0.800 | 1.000 | 0.987 | 0.5062 | 0.889 |
|  | DL-assisted junior2 | 4 | 0 | 76 | 0 | 1.000 | 1.000 | 1.000 | 1.000 | 0.8157 | 1.000 |
|  | AI | 4 | 3 | 73 | 0 | 1.000 | 0.571 | 1.000 | 0.961 | 0.5035 | 0.727 |
| 36,46 | junior1 | 6 | 8 | 26 | 0 | 1.000 | 0.429 | 1.000 | 0.765 | 0.7767 | 0.600 |
|  | junior2 | 6 | 10 | 24 | 0 | 1.000 | 0.375 | 1.000 | 0.706 | 0.4675 | 0.545 |
|  | DL-assisted junior1 | 6 | 1 | 33 | 0 | 1.000 | 0.857 | 1.000 | 0.971 | 0.8670 | 0.923 |
|  | DL-assisted junior2 | 6 | 0 | 34 | 0 | 1.000 | 1.000 | 1.000 | 1.000 | 0.7771 | 1.000 |
|  | AI | 3 | 0 | 34 | 3 | 0.919 | 1.000 | 0.500 | 1.000 | 0.6970 | 0.667 |
