# Supplementary material for: The application of deep learning in early enamel demineralization detection
Source: PeerJ. 2025 Jan 2;13:e18593. doi: 10.7717/peerj.18593 (PMC11700490; doi:10.7717/peerj.18593)
Supplement: Supplemental Information 2 [file peerj-13-18593-s002.docx]

**Recognition of each tooth position by the validation set**

| tooth position | TP | FP | TN | FN | NPV | PPV | SEN | SPEC | IoU | F1-score |
| --- | --- | --- | --- | --- | --- | --- | --- | --- | --- | --- |
| 11,12,21,22 | 27 | 9 | 123 | 1 | 0.992 | 0.750 | 0.964 | 0.932 | 0.678 | 0.844 |
| 13,23 | 13 | 3 | 61 | 2 | 0.968 | 0.813 | 0.867 | 0.953 | 0.686 | 0.839 |
| 14,15,24,25 | 19 | 0 | 131 | 0 | 1.000 | 1.000 | 1 | 1 | 0.718 | 1.000 |
| 16,26 | 10 | 3 | 63 | 1 | 0.984 | 0.769 | 0.909 | 0.955 | 0.807 | 0.833 |
| 31,32,41,42 | 3 | 5 | 150 | 2 | 0.987 | 0.375 | 0.6 | 0.968 | 0.632 | 0.462 |
| 33,43 | 8 | 1 | 69 | 1 | 0.986 | 0.889 | 0.889 | 0.986 | 0.689 | 0.889 |
| 34,35,44,45 | 19 | 0 | 141 | 0 | 1.000 | 1.000 | 1 | 1 | 0.652 | 1.000 |
| 36,46 | 2 | 6 | 72 | 0 | 1.000 | 0.250 | 1 | 0.923 | 0.660 | 0.400 |
