# Supplementary material for: The application of deep learning in early enamel demineralization detection
Source: PeerJ. 2025 Jan 2;13:e18593. doi: 10.7717/peerj.18593 (PMC11700490; doi:10.7717/peerj.18593)
Supplement: Supplemental Information 3 [file peerj-13-18593-s003.docx]

|  | maxillary teeth | | | | | mandibular teeth | | | | |
| --- | --- | --- | --- | --- | --- | --- | --- | --- | --- | --- |
| Average demineralization index | 11, 21 | 12, 22 | 13, 23 | 14，15, 24, 25 | 16,26 | 31, 41 | 32, 42 | 33, 43 | 34, 44, 35, 45 | 36, 46 |
|  | 0.1508 | 0.1793 | 0.2171 | 0.1427 | 0.2434 | 0.0752 | 0.1332 | 0.1432 | 0.1660 | 0.1542 |

**Demineralization index of each tooth position**
