# Supplementary material for: The application of deep learning in early enamel demineralization detection
Source: PeerJ. 2025 Jan 2;13:e18593. doi: 10.7717/peerj.18593 (PMC11700490; doi:10.7717/peerj.18593)
Supplement: Supplemental Information 4 [file peerj-13-18593-s004.docx]

Content

[draw box_utils.py 2](#_Toc184498506)

[my dataset coco.py 4](#_Toc184498507)

[my dataset_voc.py 5](#_Toc184498508)

[plot_curve.py 8](#_Toc184498509)

[predict.py 8](#_Toc184498510)

[README.md 10](#_Toc184498511)

[train.py 11](#_Toc184498512)

[train convnext.py 15](#_Toc184498513)

[train multi_GPU.py 18](#_Toc184498514)

[transforms.py 21](#_Toc184498515)

[validation.py 22](#_Toc184498516)

[Backbone 23](#_Toc184498517)

[feature_pyramid network.py 23](#_Toc184498518)

[resnet50 fpn model.py 24](#_Toc184498519)

[network files 26](#_Toc184498520)

[boxes.py 26](#_Toc184498521)

[det utils.py 26](#_Toc184498522)

[faster_rcnn_framework.py 29](#_Toc184498523)

[image_list.py 31](#_Toc184498524)

[mask_rcnn.py 31](#_Toc184498525)

[roi_head.py 32](#_Toc184498526)

[rpn function.py 38](#_Toc184498527)

[transform.py 41](#_Toc184498528)

[train utils 42](#_Toc184498529)

[coco_eval.py 42](#_Toc184498530)

[coco_utils.py 44](#_Toc184498531)

[distributed_utils.py 45](#_Toc184498532)

[group_by_aspect_ratio.py 47](#_Toc184498533)

[train eval utils.py 48](#_Toc184498534)

## draw box_utils.py

寻找列表中最大元素的下标
Find the index of the largest element in the list.

将目标边界框和类别信息绘制到图片上
Draw the target bounding box and category information on the image.

计算脱矿区域面积
Calculate the area of the demineralization region.

展示类别信息和概率分数
Display category information and probability scores.

展示脱矿区域面积
Display the area of the demineralization region.

匹配牙齿和脱矿区域
Match teeth with demineralization regions.

即将计算iou
About to calculate IoU.

对应的牙齿面积
Corresponding tooth area.

分割出牙齿的mask个数
Number of tooth masks segmented.

实现脱矿区域实例分割效果
Implement instance segmentation effect for demineralization regions.

实现牙齿实例分割效果
Implement instance segmentation effect for teeth.

将目标边界框信息，类别信息，mask信息绘制在图片上
Draw target bounding box information, category information, and mask information on the image.

过滤掉低概率的目标
Filter out low-probability targets.

绘制目标边界框
Draw the target bounding box.

绘制类别和概率信息
Draw category and probability information.

进行脱矿区域和牙齿匹配，返回匹配牙齿的边界框坐标和在mask2中的index
Match demineralization regions and teeth, and return the bounding box coordinates of matched teeth and their index in mask2.

计算iou完成
IoU calculation completed.

画出实例分割后的牙齿
Draw the teeth after instance segmentation.

所匹配的牙齿编号为{teethIndex}，下面开始绘制
The matched tooth indices are {teethIndex}. Now beginning to draw.

## my dataset coco.py

实例化

*English*: Instantiation

获取coco数据索引与类别名称的关系

*English*: Get the relationship between COCO data indices and category names

注意在object80中的索引并不是连续的，虽然只有80个类别，但索引还是按照stuff91来排序的

*English*: Note that the indices in object80 are not continuous. Although there are only 80 categories, the indices are sorted according to stuff91.

将缺失的类别名称设置成N/A,自己构建的就不用管，和data_classes不同的是去掉了第一个_background_

*English*: Set missing category names to "N/A". If you constructed the dataset yourself, this step can be skipped. Unlike data_classes, the first _background_ is removed.

下面也不用管

*English*: You can also skip the following.

移除没有目标，或者目标面积非常小的数据

*English*: Remove data without targets or with very small target areas.

只筛选出单个对象的情况

*English*: Only filter out cases with a single object.

就是通过调用这个函数生成mask

*English*: The mask is generated by calling this function.

筛选出合法的目标，即x_max>x_min且y_max>y_min

*English*: Filter out valid targets where xmax>xminx_{\text{max}} > x_{\text{min}} and ymax>yminy_{\text{max}} > y_{\text{min}}.

根据索引获取图片id,根据图片id可以获得该图片的annotation_id,然后coco_target是加载该图片标注信息的id

*English*: Get the image ID based on the index. Using the image ID, you can obtain the annotation ID of the image. Then, coco_target is the ID for loading the annotation information of the image.

## my dataset_voc.py

**存储图片路径**

English: Store image paths.

**存储xml文件路径**

English: Store XML file paths.

**存储解析的xml字典文件**

English: Store parsed XML dictionary files.

**存储SegmentationObject图片路径**

English: Store SegmentationObject image paths.

**存储解析的目标boxes等信息**

English: Store parsed target boxes and other information.

**存储读取的SegmentationObject图片信息**

English: Store read SegmentationObject image information.

**检查图片、xml文件以及mask是否都在**

English: Check if the images, XML files, and masks all exist.

**解析xml中bbox信息**

English: Parse bbox information from the XML.

**将xml文件解析成字典**

English: Parse the XML file into a dictionary.

**解析出目标信息**

English: Parse out target information.

**读取SegmentationObject并检查是否和bboxes信息数量一致**

English: Read SegmentationObject and check if it matches the number of bbox information.

**需要检查一下标注的bbox个数是否和instances个数一致**

English: Need to check if the number of annotated bboxes matches the number of instances.

**方便统计所有图片的高宽比例信息**

English: Facilitate the statistics of the height-to-width ratio information of all images.

**方便构建COCO()**

English: Facilitate building COCO().

**将xml文件解析成字典形式，参考tensorflow的recursive_parse_xml_to_dict**

English: Parse the XML file into a dictionary format, referencing TensorFlow's recursive_parse_xml_to_dict.

**将xml树解析到字典中**

English: Parse the XML tree into a dictionary.

**递归遍历标签信息**

English: Recursively traverse tag information.

**因为object可能有多个，所以需要放入列表里**

English: Since there may be multiple objects, they need to be put into a list.

**解析出bboxes、labels、iscrowd以及ares等信息**

English: Parse out information such as bboxes, labels, iscrowd, and areas.

**将xml解析成dict的Annotation数据**

English: Parse the XML into dictionary Annotation data.

**类别与索引对应关系**

English: The correspondence between categories and indices.

**进一步检查数据，有的标注信息中可能有w或h为0的情况，这样的数据会导致计算回归loss为nan**

English: Further check the data; some annotations may have ww or hh equal to 0, which can lead to regression loss being NaN.

**convert everything into a torch.Tensor**

English: Convert everything into a torch.Tensor.

## plot_curve.py

设置横坐标整数间隔
Set the x-axis to integer intervals

防止出现保存图片显示不全的情况
 Prevent the saved image from being cropped

## predict.py

不包含背景
Excluding background

请输入jpg或JPG格式的图片
Please input an image in jpg or JPG format

using {} device.
Using {} device.

文件不存在
The file does not exist

json 文件 {} 不存在
The json file {} does not exist

原始图片
Original image

保存成功
Successfully saved

## README.md

口内照脱矿项目
Intraoral Demineralization Project

口内照脱矿项目使用Mask R-CNN对牙齿脱矿区域进行识别，并使用segment-anything对整张图片进行自动分割。
The Intraoral Demineralization Project uses Mask R-CNN to identify demineralized areas of teeth and uses segment-anything for automatic segmentation of the entire image.

通过识别到的脱矿区域来计算segment-anything识别到的mask的IOU，从而确定脱矿区域所在的牙齿蒙版，最后计算面积比输出在图片上。
The demineralized areas identified are used to calculate the IOU of the mask identified by segment-anything, which determines the tooth mask containing the demineralized region, and finally, the area ratio is calculated and displayed on the image.

项目安装分为两个部分：requirements.txt和segment-anything模块
The project installation is divided into two parts: requirements.txt and the segment-anything module

参数解释：
Parameter explanation:

--image：单个图片路径，必须传

--image: The path of a single image, which is required

--model：模型权重文件地址，*default*='./save_weights/model_best.pth'

--model: The model weight file path, *default*='./save_weights/model_best.pth'

--save_dir：输出图片位置，*default*='./result'

--save_dir: The output image directory, *default*='./result'

--seg_model：segment_anything模型权重，*default*='./seg_model/sam_vit_h_4b8939.pth'

--seg_model: The segment_anything model weight, *default*='./seg_model/sam_vit_h_4b8939.pth'

项目是把原始数据集转换成COCO数据集格式进行训练
The project converts the original dataset into the COCO dataset format for training

## train.py

如果GPU显存很小，batch_size不能设置很大，建议将norm_layer设置成FrozenBatchNorm2d(默认是nn.BatchNorm2d)

If the GPU memory is small, batch_size should not be set too large. It is recommended to set the norm_layer to FrozenBatchNorm2d (the default is nn.BatchNorm2d).

FrozenBatchNorm2d的功能与BatchNorm2d类似，但参数无法更新

FrozenBatchNorm2d functions similarly to BatchNorm2d, but its parameters cannot be updated.

trainable_layers包括['layer4', 'layer3', 'layer2', 'layer1', 'conv1']， 5代表全部训练

trainable_layers includes ['layer4', 'layer3', 'layer2', 'layer1', 'conv1'], where 5 represents training all layers.

用来保存coco_info的文件,目标检测和实例分割

File used to save coco_info, for object detection and instance segmentation.

数据集的存放位置

The location where the dataset is stored.

通过此操作得到的数据已经是经过data_transform的数据了

The data obtained through this operation has already been processed by data_transform.

是否按图片相似高宽比采样图片组成batch

Whether to sample images based on similar aspect ratios to form the batch.

使用的话能够减小训练时所需GPU显存，默认使用

If used, it can reduce the GPU memory required during training, and it is used by default.

注意这里的collate_fn是自定义的，因为读取的数据包括image和targets，不能直接使用默认的方法合成batch

Note that the collate_fn here is customized, as the data read includes images and targets, so the default method cannot be used to form a batch.

训练设备类型

Type of training device.

训练数据集的根目录

Root directory of the training dataset.

检测目标类别数(不包含背景)

The number of object classes (excluding the background).

文件保存地址

File save location.

若需要接着上次训练，则指定上次训练保存权重文件地址

If you need to continue from the last training, specify the path to the saved weights from the previous training.

指定接着从哪个epoch数开始训练

Specify from which epoch to continue training.

训练的总epoch数

The total number of epochs for training.

学习率

Learning rate.

SGD的momentum参数

Momentum parameter for SGD.

SGD的weight_decay参数

Weight decay parameter for SGD.

针对torch.optim.lr_scheduler.MultiStepLR的参数

Parameters for torch.optim.lr_scheduler.MultiStepLR.

针对torch.optim.lr_scheduler.MultiStepLR的参数

Parameters for torch.optim.lr_scheduler.MultiStepLR.

训练的batch size(如果内存/GPU显存充裕，建议设置更大)

Batch size for training (if memory/GPU memory is sufficient, it is recommended to set it larger).

是否使用混合精度训练(需要GPU支持混合精度)

Whether to use mixed precision training (requires GPU support for mixed precision).

## train convnext.py

如果GPU显存很小，batch_size不能设置很大，建议将norm_layer设置成FrozenBatchNorm2d(默认是nn.BatchNorm2d)

If the GPU memory is small, the batch_size should not be set too large. It is recommended to set the norm_layer to FrozenBatchNorm2d (the default is nn.BatchNorm2d).

FrozenBatchNorm2d的功能与BatchNorm2d类似，但参数无法更新

FrozenBatchNorm2d functions similarly to BatchNorm2d, but its parameters cannot be updated.

trainable_layers包括['layer4', 'layer3', 'layer2', 'layer1', 'conv1']， 5代表全部训练

trainable_layers includes ['layer4', 'layer3', 'layer2', 'layer1', 'conv1'], and 5 represents training all layers.

数据集的存放位置

The location where the dataset is stored.

通过此操作得到的数据已经是经过data_transform的数据了

The data obtained through this operation has already been processed by data_transform.

是否按图片相似高宽比采样图片组成batch

Whether to sample images based on similar aspect ratios to form a batch.

使用的话能够减小训练时所需GPU显存，默认使用

If used, it can reduce the GPU memory required during training. It is used by default.

如果按照图片高宽比采样图片，dataloader中需要使用batch_sampler

If sampling images based on aspect ratio, the dataloader needs to use batch_sampler.

注意这里的collate_fn是自定义的，因为读取的数据包括image和targets，不能直接使用默认的方法合成batch

Note that the collate_fn here is custom because the data read includes images and targets, and the default method cannot be used to batch them directly.

数据集的存放位置

The location where the dataset is stored.

把val删了

Delete the val.

训练设备类型

Type of training device.

训练数据集的根目录

Root directory of the training dataset.

检测目标类别数(不包含背景)

Number of detection classes (excluding background).

文件保存地址

File save location.

若需要接着上次训练，则指定上次训练保存权重文件地址

If resuming from the previous training, specify the location of the saved weights file from the previous training.

指定接着从哪个epoch数开始训练

Specify from which epoch to resume training.

训练的总epoch数

Total number of epochs for training.

学习率

Learning rate.

SGD的momentum参数

Momentum parameter for SGD.

SGD的weight_decay参数

Weight decay parameter for SGD.

针对torch.optim.lr_scheduler.MultiStepLR的参数

Parameters for torch.optim.lr_scheduler.MultiStepLR.

训练的batch size(如果内存/GPU显存充裕，建议设置更大)

Batch size for training (if memory/GPU is sufficient, it is recommended to set a larger value).

是否使用混合精度训练(需要GPU支持混合精度)

Whether to use mixed-precision training (requires GPU support for mixed precision).

检查保存权重文件夹是否存在，不存在则创建

Check if the folder for saving weights exists; if not, create it.

## train multi_GPU.py

如果GPU显存很小，batch_size不能设置很大，建议将norm_layer设置成FrozenBatchNorm2d(默认是nn.BatchNorm2d)

If the GPU memory is small, the batch_size should not be set too large. It is recommended to set the norm_layer to FrozenBatchNorm2d (the default is nn.BatchNorm2d).

FrozenBatchNorm2d的功能与BatchNorm2d类似，但参数无法更新

FrozenBatchNorm2d functions similarly to BatchNorm2d, but its parameters cannot be updated.

trainable_layers包括['layer4', 'layer3', 'layer2', 'layer1', 'conv1']， 5代表全部训练

trainable_layers includes ['layer4', 'layer3', 'layer2', 'layer1', 'conv1'], and 5 represents training all layers.

训练文件的根目录(coco2017)

Root directory of the training files (coco2017).

训练设备类型

Type of training device.

检测目标类别数(不包含背景)

Number of detection classes (excluding background).

每块GPU上的batch_size

Batch size per GPU.

指定接着从哪个epoch数开始训练

Specify from which epoch to resume training.

训练的总epoch数

Total number of epochs for training.

数据加载以及预处理的线程数

Number of data loading and preprocessing threads.

学习率，这个需要根据gpu的数量以及batch_size进行设置0.02 / bs * num_GPU

Learning rate, which should be set based on the number of GPUs and batch size (0.02 / batch size * number of GPUs).

SGD的momentum参数

Momentum parameter for SGD.

SGD的weight_decay参数

Weight decay parameter for SGD.

针对torch.optim.lr_scheduler.StepLR的参数

Parameters for torch.optim.lr_scheduler.StepLR.

针对torch.optim.lr_scheduler.MultiStepLR的参数

Parameters for torch.optim.lr_scheduler.MultiStepLR.

训练过程打印信息的频率

Frequency of printing information during training.

文件保存地址

File save location.

基于上次的训练结果接着训练

Resume training from the previous training result.

开启的进程数(注意不是线程)

Number of processes to be used (note, not threads).

是否使用混合精度训练(需要GPU支持混合精度)

Whether to use mixed precision training (requires GPU support for mixed precision).

如果指定了保存文件地址，检查文件夹是否存在，若不存在，则创建

If a save file location is specified, check if the folder exists; if not, create it.

## transforms.py

组合多个transform函数

Combine multiple transform functions.

将PIL图像转为Tensor

Convert PIL image to Tensor.

随机水平翻转图像以及bboxes

Randomly flip image and bounding boxes horizontally.

翻转对应bbox坐标信息

Flip corresponding bounding box coordinate information.

## validation.py

该脚本用于调用训练好的模型权重去计算验证集/测试集的COCO指标

This script is used to call the trained model weights to calculate COCO metrics for the validation/test dataset.

以及每个类别的mAP(IoU=0.5)

And the mAP (IoU=0.5) for each category.

注意这里的collate_fn是自定义的，因为读取的数据包括image和targets，不能直接使用默认的方法合成batch

Note that the collate_fn here is custom because the data includes images and targets, so the default method cannot be used to form batches.

载入你自己训练好的模型权重

Load your own trained model weights.

使用设备类型

Use device type.

检测目标类别数(不包含背景)

Number of detection classes (excluding background).

数据集的根目录

The root directory of the dataset.

训练好的权重文件

Trained weight file.

类别索引和类别名称对应关系

The correspondence between category index and category name.

# Backbone

## feature_pyramid network.py

遍历模型子模块按顺序存入有序字典
Traverse the submodules of the model in order and store them in an ordered dictionary

只保存layer4及其之前的结构，舍去之后不用的结构
Only retain the structure of layer4 and the previous layers, discarding the unused ones after that

依次遍历模型的所有子模块，并进行正向传播
Traverse all the submodules of the model sequentially and perform forward propagation

收集layer1, layer2, layer3, layer4的输出
Collect the outputs of layer1, layer2, layer3, and layer4

用来调整resnet特征矩阵(layer1,2,3,4)的channel（kernel_size=1）
Used to adjust the channels of the ResNet feature maps (layer1, 2, 3, 4) (kernel_size=1)

对调整后的特征矩阵使用3x3的卷积核来得到对应的预测特征矩阵
Use a 3x3 convolutional kernel on the adjusted feature maps to obtain the corresponding predicted feature maps

将resnet layer4的channel调整到指定的out_channels
Adjust the channels of ResNet layer4 to the specified out_channels

result中保存着每个预测特征层
The result contains each predicted feature layer

将layer4调整channel后的特征矩阵，通过3x3卷积后得到对应的预测特征矩阵
The feature map adjusted from layer4 channels is passed through a 3x3 convolution to obtain the corresponding predicted feature map

在layer4对应的预测特征层基础上生成预测特征矩阵5
Generate the predicted feature map 5 based on the predicted feature map of layer4

## resnet50 fpn model.py

搭建resnet50_fpn——backbone
Build resnet50_fpn backbone

resnet50的预训练权重，如果不使用就默认为空
Pretrained weights of resnet50, defaults to empty if not used

默认是nn.BatchNorm2d，如果GPU显存很小，batch_size不能设置很大，建议将norm_layer设置成FrozenBatchNorm2d(默认是nn.BatchNorm2d)
Default is nn.BatchNorm2d. If GPU memory is small and batch_size cannot be large, it is recommended to set norm_layer to FrozenBatchNorm2d (default is nn.BatchNorm2d)

指定训练哪些层结构
Specify which layers to train

指定哪些层的输出需要返回
Specify which layers' outputs need to be returned

在输出的特征层基础上额外添加的层结构
Additional layers added on top of the output feature layers

载入预训练权重
Load pretrained weights

如果要训练所有层结构的话，不要忘了conv1后还有一个bn1
If training all layers, don't forget that there is also a bn1 after conv1

只训练不在layers_to_train列表中的层结构
Only train layers that are not in the layers_to_train list

返回的特征层个数肯定大于0小于5
The number of returned feature layers must be greater than 0 and less than 5

# network files

## boxes.py

移除宽高小于指定阈值的索引
Remove boxes which contain at least one side smaller than min_size

裁剪预测的boxes信息，将越界的坐标调整到图片边界上
Clip predicted boxes information and adjust out-of-bounds coordinates to image boundaries

限制x坐标范围在[0,width]之间
Limit the x-coordinate range to [0, width]

限制y坐标范围在[0,height]之间
Limit the y-coordinate range to [0, height]

## det utils.py

遍历每张图像的matched_idxs
 Iterate through the matched_idxs for each image.

= 1的为正样本, nonzero返回非零元素索引
 Values >= 1 are positive samples, nonzero returns the indices of non-zero elements.

= 0的为负样本
 Values = 0 are negative samples.

如果正样本数量不够就直接采用所有正样本
 If there are not enough positive samples, use all the positive samples.

如果负样本数量不够就直接采用所有负样本
 If there are not enough negative samples, use all the negative samples.

随机选择指定数量的正负样本
 Randomly select the specified number of positive and negative samples.

创建蒙版
 Create binary mask.

正样本和负样本蒙版为1
 Positive and negative sample masks are set to 1.

结合anchors和与之对应的gt计算regression参数
 Combine anchors with corresponding gt to calculate regression parameters.

统计每张图像的anchors个数，方便后面拼接在一起处理后在分开
 Count the number of anchors for each image to facilitate processing together and splitting later.

anchors的宽度和高度
 Width and height of the anchors.

anchor中心点坐标
 Center point coordinates of the anchors.

RPN中为[1,1,1,1], fastrcnn中为[10,10,5,5]
 In RPN, it is [1, 1, 1, 1], in Faster R-CNN, it is [10, 10, 5, 5].

预测anchors/proposals的中心坐标x回归参数
 Regression parameters for predicting the center x-coordinate of anchors/proposals.

预测anchors/proposals的中心坐标y回归参数
 Regression parameters for predicting the center y-coordinate of anchors/proposals.

预测anchors/proposals的宽度回归参数
 Regression parameters for predicting the width of anchors/proposals.

预测anchors/proposals的高度回归参数
 Regression parameters for predicting the height of anchors/proposals.

限制
 Limit.

得到生成的proposal的xmin, ymin, xmax, ymax
 Obtain the generated proposal's xmin, ymin, xmax, ymax.

计算anchors与每个gtboxes匹配的iou最大值，并记录索引，
 Calculate the maximum IoU value of each anchor with the ground truth boxes and record the index.

iou小于low_threshold的索引
 The index of IoUs less than low_threshold.

iou在low_threshold与high_threshold之间的索引值
 The index values where IoU is between low_threshold and high_threshold.

是否启用第一条正样本匹配准则(当iou<0.7但为该类的最大iou时设置为正样本)
 Whether to enable the first positive sample matching criterion (when IoU < 0.7 but it is the highest IoU for that class, set it as a positive sample).

对于每个gt boxes寻找与其iou最大的anchor，
 For each ground truth box, find the anchor with the highest IoU.

寻找每个gt boxes与其iou最大的anchor索引，一个gt匹配到的最大iou可能有多个anchor
 Find the anchor index with the highest IoU for each ground truth box. A ground truth may match multiple anchors with the highest IoU.

保留该anchor匹配gt最大iou的索引，即使iou低于设定的阈值
 Keep the index of the anchor that matches the ground truth with the highest IoU, even if the IoU is below the set threshold.

## faster_rcnn_framework.py

Here are the original Chinese comments and their translations without the dashes:

加背景 - Add background

预处理resize时限制的最小尺寸与最大尺寸 - The minimum and maximum size limits during preprocessing resizing

预处理normalize时使用的均值和方差 - Mean and standard deviation used for preprocessing normalization

rpn中在nms处理前保留的proposal数(根据score) 这个前后相等是针对带fpn的网络,不同层都生成2000个anchor最后总的仍取2000 - The number of proposals to keep before NMS processing in RPN (based on score). This is equal for networks with FPN, where 2000 anchors are generated for each layer, and finally, 2000 are selected in total

rpn中在nms处理后保留的proposal数 - The number of proposals to keep after NMS processing in RPN

rpn计算损失时，采集正负样本设置的阈值 - The threshold for collecting positive and negative samples during RPN loss calculation

rpn计算损失时采样的样本数，以及正样本占总样本的比例 - The number of samples sampled during RPN loss calculation and the proportion of positive samples in the total samples

Box parameters - Box parameters

移除低目标概率 - Remove low-probability targets

fast rcnn中进行nms处理的阈值 - The NMS threshold for Fast R-CNN processing

对预测结果根据score排序取前100个目标 - Sort the prediction results by score and select the top 100 targets

fast rcnn计算误差时，采集正负样本设置的阈值 - The threshold for collecting positive and negative samples during Fast R-CNN error calculation

fast rcnn计算误差时采样的样本数，以及正样本占所有样本的比例 - The number of samples sampled during Fast R-CNN error calculation and the proportion of positive samples in all samples

roi align - ROI Align

roi pooling后的展平处理两个全连接层部分 - Flattening the processed ROI after pooling and applying two fully connected layers

将roi pooling, box_head以及box_predictor结合在一起 - Combine ROI pooling, box_head, and box_predictor together

**对数据进行标准化，缩放，打包成batch等处理部分** - Processing part such as data normalization, scaling, and batching.

## image_list.py

padding后的图像数据 - Padded image data

padding前的图像尺寸 - Original image sizes before padding

## mask_rcnn.py

有序字典 - Ordered dictionary

传给父类,完成nn.Sequential的创建 - Pass to the parent class to complete the creation of nn.Sequential

## roi_head.py

计算类别损失信息 正样本的标签就是对应的gt_box的标签, 负样本的标签就是0

Calculate the classification loss information. The label of the positive sample is the label corresponding to the gt_box, and the label of the negative sample is 0.

返回标签类别大于0的索引

Returns the index where the label category is greater than 0.

返回标签类别大于0位置的类别信息

Returns the category information at the positions where the label category is greater than 0.

计算边界框损失信息

Calculate the bounding box loss information.

从CNN的结果中，通过取对应于具有最大概率类别的掩码（这些掩码的大小是固定的，并且直接由CNN输出）来后处理掩码，并将掩码返回在BoxList的掩码字段中。

Post-process the masks from the results of the CNN by taking the masks corresponding to the classes with the maximum probability (which are of fixed size and directly output by the CNN), and return the masks in the mask field of the BoxList.

给定分割掩码和对应于图像中掩码位置的边界框，该函数裁剪和调整掩码的大小，以准备掩码被送入损失计算作为目标。

Given segmentation masks and the bounding boxes corresponding to the location of the masks in the image, this function crops and resizes the masks to prepare them to be fed to the loss computation as targets.

计算预测掩码与真实gt掩码之间的BCELoss

Calculate the BCELoss between the predicted mask and the real gt mask.

将gt_boxes拼接到proposal后面

Concatenate gt_boxes to the proposal.

为每个proposal匹配对应的gt_box，并划分到正负样本中

Match each proposal with the corresponding gt_box and divide it into positive and negative samples.

BalancedPositiveNegativeSampler

Balanced Positive Negative Sampler.

按给定数量和比例采样正负样本

Sample positive and negative samples in a given quantity and ratio.

将gt_boxes拼接到proposal后面，因为此时正样本的数量很少，所以将gt_box也添加到proposal当中

Concatenate gt_boxes to the proposal because the number of positive samples is very small at this time, so gt_box is also added to the proposal.

为每个proposal匹配对应的gt_box，并划分到正负样本中

Match each proposal with the corresponding gt_box and divide it into positive and negative samples.

根据gt和proposal计算边框回归参数（针对gt的）

Calculate the bounding box regression parameters based on gt and proposal (for gt).

对网络的预测数据进行后处理，包括

Post-process the predicted data of the network, including:

根据proposal以及预测的回归参数计算出最终bbox坐标

Calculate the final bbox coordinates based on the proposal and the predicted regression parameters.

对预测类别结果进行softmax处理

Perform softmax processing on the predicted category results.

裁剪预测的boxes信息，将越界的坐标调整到图片边界上

Crop the predicted boxes information and adjust the out-of-boundary coordinates to the image boundary.

移除所有背景信息

Remove all background information.

移除低概率目标

Remove low-probability targets.

移除小尺寸目标

Remove small-sized targets.

执行nms处理，并按scores进行排序

Perform nms processing and sort by scores.

根据scores排序返回前topk个目标

Return the top k targets sorted by scores.

检查targets的数据类型是否正确

Check if the data type of targets is correct.

划分正负样本，统计对应gt的标签以及边界框回归信息

Divide positive and negative samples, and count the corresponding gt labels and bounding box regression information.

将采集样本通过Multi-scale RoIAlign pooling层

Pass the collected samples through the Multi-scale RoIAlign pooling layer.

通过roi_pooling后的两层全连接层

Through the two fully connected layers after roi_pooling.

接着分别预测目标类别和边界框回归参数

Then predict the target category and bounding box regression parameters separately.

训练模式的时候result就是一个空列表, 预测模式是boxes, labels, scores

In training mode, the result is an empty list; in prediction mode, it is boxes, labels, scores.

在这里选择在0,1,2,3特征层上进行池化, 符合论文中所说的

Here, choose to perform pooling on feature layers 0, 1, 2, 3, as stated in the paper.

## rpn function.py

anchors生成器

Anchor generator

模块支持在多个特征图上计算多个尺寸和纵横比的anchors。

The module supports computing anchors at multiple sizes and aspect ratios per feature map.

sizes和aspect_ratios应该有相同数量的元素，并且应该对应于特征图的数量。

sizes and aspect_ratios should have the same number of elements, and it should correspond to the number of feature maps.

AnchorGenerator将输出每空间位置feature map i的sizes[i] * aspect_ratios[i]个anchors。

AnchorGenerator will output a set of sizes[i] * aspect_ratios[i] anchors per spatial location for feature map i.

计算anchor大小

Compute anchor sizes

生成的anchors模板都是以（0, 0）为中心的，shape [len(ratios)*len(scales), 4]

The generated anchor templates are centered at (0, 0), shape [len(ratios)*len(scales), 4]

计算预测特征图对应原始图像上的所有anchors的坐标

Compute the coordinates of all anchors on the original image corresponding to the prediction feature map

将计算得到的所有anchors信息进行缓存

Cache all calculated anchor information

通过滑动窗口计算预测目标概率与bbox regression参数

Calculate the predicted target probability and bbox regression parameters through the sliding window

调整tensor顺序，并进行reshape

Adjust the tensor order and perform reshape

对box_cla和box_regression两个list中的每个预测特征层的预测信息的tensor排列顺序以及shape进行调整 -> [N, -1, C]

Adjust the tensor arrangement order and shape for each prediction feature layer's prediction information in the box_cla and box_regression lists -> [N, -1, C]

计算每个anchors最匹配的gt，并划分为正样本，背景以及废弃的样本

Calculate the most matching gt for each anchor and divide it into positive samples, background, and discarded samples

筛除小boxes框，nms处理，根据预测概率获取前post_nms_top_n个目标

Filter out small boxes, perform nms processing, and get the top post_nms_top_n targets based on prediction probability

计算RPN损失，包括类别损失（前景与背景），bbox regression损失

Calculate RPN loss, including category loss (foreground and background), bbox regression loss

## transform.py

标准化处理 此时的数据格式是tensor格式

Standardization processing, the data format is tensor at this time.

将图片缩放到指定的大小范围内，并对应缩放bboxes信息

Scale the image to the specified size range, and correspondingly scale the bboxes information.

如果是训练模式，则指定输入图片的最小边长，注意是self.min_size不是min_size

If it is training mode, specify the minimum side length of the input image, note that it is self.min_size, not min_size.

指定输入图片的最小边长，注意是self.min_size不是min_size

Specify the minimum side length of the input image, note that it is self.min_size, not min_size.

将一批图像打包成一个batch返回（注意batch中每个tensor的shape是相同的）显然要相同

Pack a batch of images into a batch and return (note that the shape of each tensor in the batch is the same) obviously needs to be the same.

对网络的预测结果进行后处理（主要将bboxes还原到原图像尺度上）

Post-process the network's prediction results (mainly restore bboxes to the original image scale).

将boxes参数根据图像的缩放情况进行相应缩放

Scale the boxes parameter according to the scaling situation of the image.

# train utils

## coco_eval.py

将多个进程之间的数据汇总在一起

Merge data between multiple processes

去除重复的图片索引，多GPU训练时为了保证每个进程的训练图片数量相同，可能将一张图片分配给多个进程

Remove duplicate image indices. During multi-GPU training, to ensure that each process has the same number of training images, an image may be assigned to multiple processes.

将预测的结果转换成COCOeval指定的格式，针对目标检测任务

Convert the predicted results into the format specified by COCOeval for object detection tasks.

对于coco_eval, 需要的每个box的数据格式为[x_min, y_min, w, h] 而我们预测的box格式是[x_min, y_min, x_max, y_max]，所以需要转下格式

For coco_eval, the format required for each box is [x_min, y_min, w, h], but our predicted box format is [x_min, y_min, x_max, y_max], so we need to convert the format.

防止出现重复的数据

Prevent duplicate data

将预测的结果转换成COCOeval指定的格式，针对实例分割任务

Convert the predicted results into the format specified by COCOeval for instance segmentation tasks.

同步所有进程中的数据

Synchronize data from all processes

主进程上保存即可

Save on the main process is sufficient.

只在主进程上评估即可

Evaluation is only needed on the main process.

## coco_utils.py

删除coco数据集中没有目标，或者目标面积非常小的数据

Delete images from the COCO dataset that have no objects or objects with very small areas.

如果它为空，则表示没有标注

If it's empty, there is no annotation.

如果所有框的面积都接近零，则表示没有标注

If all boxes have close to zero area, there is no annotation.

如果有有效的标注

If there is a valid annotation.

将COCO多边形掩码转换为二进制掩码

Convert COCO polygon masks to binary masks.

如果mask为空，则说明没有目标，直接返回数值为0的mask

If the mask is empty, it means there is no object, and return a mask with all values being 0.

## distributed_utils.py

deque简单理解成加强版list

deque can be simply understood as an enhanced version of list.

收集各个进程中的数据

Collect data from all processes.

单GPU的情况

Single GPU situation.

多GPU的情况

Multi-GPU situation.

如果attr在self.meters中

If attr is in self.meters.

如果attr在self.__dict__中

If attr is in self.__dict__.

总共用时

Total time.

这一步的时间

Time for this step.

数据加载时间

Data loading time.

检查是否支持分布式环境

Check if distributed environment is supported.

进程数

Number of processes.

这不是使用分布式模式

Not using distributed mode.

禁用当不在主进程

Disabled when not in the master process.

## group_by_aspect_ratio.py

计算所有数据集中的图片width/height比例

Calculate the width/height ratio of all images in the dataset.

将[0.5, 2]区间划分成2*k+1等份

Divide the interval [0.5, 2] into 2*k+1 equal parts.

统计所有图像比例在bins区间中的位置索引

Count the position indices of all image ratios in the bins interval.

统计每个区间的频次

Count the frequency of each interval.

使用作为宽高比量化的bins

Using as bins for aspect ratio quantization.

每个区间的实例数量

The number of instances per bin.

## train eval utils.py

当训练第一轮（epoch=0）时，启用warmup训练方式，可理解为热身训练

When training the first round (epoch=0), enable the warmup training method, which can be understood as a warm-up training.

混合精度训练上下文管理器，如果在CPU环境中不起任何作用

Mixed precision training context manager, which does nothing if in a CPU environment.

reduce losses over all GPUs for logging purpose

Reduce losses over all GPUs for logging purposes.

记录训练损失

Record training loss.

当计算的损失为无穷大时停止训练

Stop training when the calculated loss is infinite.

同步所有进程中的数据

Synchronize data from all processes.
